# Piperine Suppresses Pyroptosis and Interleukin-1β Release upon ATP Triggering and Bacterial Infection

**DOI:** 10.3389/fphar.2016.00390

**Published:** 2016-10-20

**Authors:** Yi-Dan Liang, Wen-Jing Bai, Chen-Guang Li, Li-Hui Xu, Hong-Xia Wei, Hao Pan, Xian-Hui He, Dong-Yun Ouyang

**Affiliations:** ^1^Department of Immunobiology, College of Life Science and Technology, Jinan UniversityGuangzhou, China; ^2^Department of Cell Biology, College of Life Science and Technology, Jinan UniversityGuangzhou, China

**Keywords:** piperine, inflammasome activation, interleukin-1β, pyroptosis, AMP-activated protein kinase

## Abstract

Piperine is a phytochemical present in black pepper (*Piper nigrum* Linn) and other related herbs, possessing a wide array of pharmacological activities including anti-inflammatory effects. Previously, we demonstrated that piperine has therapeutic effects on bacterial sepsis in mice, but the underlying mechanism has not been fully elucidated. In this study, we aimed to investigate the influences of piperine on pyroptosis in murine macrophages. The results showed that piperine dose-dependently inhibited ATP-induced pyroptosis, thereby suppressing interleukin-1β (IL-1β) or high mobility group box-1 protein (HMGB1) release in LPS-primed bone marrow-derived macrophages and J774A.1 cells. Accompanying this, ATP-induced AMP-activated protein kinase (AMPK) activation was greatly suppressed by piperine, whereas AMPK agonist metformin counteracted piperine’s inhibitory effects on pyroptosis. Moreover, piperine administration greatly reduced both peritoneal and serum IL-1β levels in the mouse model intraperitoneally infected with *Escherichia coli*, suggestive of suppressing systemic inflammation and pyroptosis. Our data indicated that piperine could protect macrophages from pyroptosis and reduced IL-1β and HMGB1 release by suppressing ATP-induced AMPK activation, suggesting that piperine may become a potential therapeutic agent against bacterial sepsis.

## Introduction

Piperine is an alkaloid present in black pepper (*Piper nigrum* Linn) and other related herbs (Wattanathorn et al., 2008). This alkaloid has been reported to possess a broad spectrum of pharmacological activities. It is well known for its anti-depressive and anti-epileptic activities (Pal et al., 2011; Mao et al., 2014). It is also known as a booster for promoting bioavailability of other drugs thus enhancing their pharmacological effects (Johnson et al., 2011; Di et al., 2015). Interestingly, piperine has been demonstrated to be a potential agent with anti-obesity (BrahmaNaidu et al., 2014), anti-gastric ulcer (Bai and Xu, 2000), anti-acute pancreatitis (Bae G. S. et al., 2011), and anti-arthritis (Murunikkara et al., 2012; Ying et al., 2013) properties. Moreover, piperine is also effective for the treatment of diarrhea (Mehmood and Gilani, 2010) and endotoxin-induced septic shock in mice (Bae et al., 2010). Therefore, piperine may be generally regarded as an anti-inflammatory agent against various inflammatory disorders as a consequence of bacterial infections or autoimmune responses.

Recently, we have demonstrated that piperine administration reduces mouse mortality, and alleviates their internal organ damages upon bacterial infection (Pan et al., 2015). One potential mechanism is that piperine treatment promotes amino acid metabolism and thus enhances mTORC1 signaling in peritoneal resident macrophages. The functions of the peritoneal macrophages are greatly enhanced in terms of their bacterial phagocytic ability and their cytokine secretion ability upon inflammatory stimulation (Pan et al., 2015). However, it is still unclear how piperine prevents internal organs from injury under the circumstance of systemic inflammatory responses during bacterial sepsis.

One consequence of bacterial infection is inflammasome activation. The inflammasome is a multiple protein complex and its activation represents the first line of innate defense against bacterial infection (Lamkanfi and Dixit, 2014; Wegiel et al., 2014). The activation of inflammasome requires two signals. First, the innate immune cells is primed by recognizing the pathogen-associated molecular patterns (PAMPs) expressed on the pathogen through their pattern recognition receptors (PRRs), resulting in the expression of critical components of inflammasome, such as nucleotide and oligomerization domain, leucine-rich repeat containing protein family, pyrin containing domain 3 (NLRP3) and pro-interleukin-1β (pro-IL-1β). Second, the inflammasome is assembled in the PAMP-primed cells upon further stimulation by damage-associated molecular patterns (DAMPs) such as ATP, culminating in recruitment of the apoptosis-associated speck-like protein containing CARD (ASC) adaptor protein. Consequently, pro-caspase-1 is activated by the inflammasome to produce the active caspase-1, which further converts pro-IL-1β into mature form IL-1β (Lamkanfi and Dixit, 2014). The latter is a potent endogenous pyrogen that promotes an increase in body temperature as well as mediating inflammatory responses. Beyond the release of mature IL-1β, one prominent consequence of inflammasome activation is pyroptosis—an inflammatory programmed cell death, which is dependent on the activation of inflammatory caspase-1 or caspase-11. Activated caspase-1 or caspase-11 can cleave the gasdermin D to release its N-terminal fragment which is critical for pyroptosis (Shi et al., 2014; Kayagaki et al., 2015). Therefore, induction of pyroptosis requires both PAMP and DAMP stimulation, as having been elegantly evaluated recently (Cullen et al., 2015), constituting the canonical inflammasome signaling. In non-canonical inflammasome signaling, lipopolysaccharide (LPS), upon penetrating into the cell, directly binds caspase-11 and activates it, leading to caspase-1 activation and pyroptosis (Shi et al., 2014; Kayagaki et al., 2015).

Many studies have indicated that inflammasome activation and pyroptosis provide protection against bacterial infection (Ceballos-Olvera et al., 2011) and experimental colitis (Zaki et al., 2010; Demon et al., 2014; Oficjalska et al., 2015). Without the protection of inflammasome mechanism due to lack of caspase-1 and caspase-11 genes, mice are vulnerable to intracellular bacterial infection (Maltez et al., 2015). However, increasing evidence has indicated that pyroptosis may be a major cause that leads to multiple organ failure and septic death (Masters et al., 2012; Wree et al., 2014). In support of this notion, mice are resistant to bacterial-induced death when the pyroptotic mechanism is lost due to caspase-11 and gasdermin D deficiency (Kayagaki et al., 2015). Although it has once believed that ‘cytokine storm’ is the main cause of sepsis, recent studies have proved that septic death can still take place in mice lacking caspase-1 activation and IL-1β production (due to caspase-1 gene deletion but retaining of caspase-11 gene), reinforcing the idea that caspase-11-mediated pyroptosis is critical for septic shock (Kayagaki et al., 2013). However, the release of IL-1β may still be regarded as a marker of pyroptosis, as one recent study has proved that it can only be released by pyroptotic cells instead of viable ones (Cullen et al., 2015).

In this study, we aimed to investigate whether piperine could suppress pyroptosis in macrophages. By using *in vitro* cell models where mouse bone marrow-derived macrophages (BMDMs) and J774A.1 cells were primed with LPS (a Gram-negative bacterial PAMP) followed by triggering with extracellular ATP [a DAMP released by hosts or bacteria (Mempin et al., 2013; Ren et al., 2014; Wegiel et al., 2014)], we found that piperine treatment significantly suppressed ATP-induced pyroptosis, which was associated with suppression of AMPK activity. Piperine administration markedly reduced IL-1β levels in the peritoneal lavage fluids and serum of mice with bacterial sepsis as compared with vehicle, indicating attenuation of systemic inflammation in the circumstance of bacterial sepsis. Our data suggest that piperine may be used to prevent bacterial sepsis by suppression of pyroptosis.

## Materials and Methods

### Reagents and Animals

Propidium iodide (PI), dimethyl sulfoxide (DMSO), Hoechst 33342, adenosine triphosphate (ATP) (P8232) and LPS (from *Escherichia coli* O111:B4) (L4391) were purchased from Sigma-Aldrich (St. Louis, MO, USA). Piperine was purchased from Guangdong Institute for Drug Control (Guangzhou, China), dissolved in DMSO and stored at -20°C. DMEM, fetal bovine serum (FBS), penicillin and streptomycin were products of ThermoFisher/Gibco (Carlsbad, CA, USA). Metformin was obtained from MedChem Express (Princeton, NJ, USA), dissolved in DMEM at 300 mM and stored at -20°C. The antibody to NLRP3 (AG-20B-0014) was purchased from Adipogen AG (Liestal, Switzerland). Antibodies against caspase-1p10 (sc-514) and actin (sc-1616-R) were obtained from Santa Cruz (Santa Cruz Biotechnology, Dallas, TX, USA). Antibodies against IL-1β (#12242), HMGB1 (#3935), AMPKα (#5832), phospho(p)-AMPKα(Thr172) (#2535), p70S6K (#2708), p-p70S6K(Thr389) (#9234), caspase-3 (#9662), β-tubulin (#2128) and horseradish peroxidase (HRP)-conjugated goat anti-mouse/-rabbit/-rat IgG were bought from Cell Signaling Technology (Danvers, MA, USA).

Female C57BL/6 mice were bought from the Experimental Animal Center of Southern Medical University (Guangzhou, China). Animal experiments were designed following the National Institutes of Health guidelines and were approved by the Committee on the Ethics of Animal Experiments of Jinan University.

### Cell Line and Cell Culture

The J774A.1 cells was obtained from the Kunming Cell Bank of Type Culture Collection, Chinese Academy of Sciences (Kunming, China) and maintained in DMEM supplemented with 10% FBS, 100 U/ml penicillin, 100 μg/ml streptomycin, and 2 mM _L_-glutamine (DMEM complete medium) at 37°C in a humidified incubator of 5% CO_2_. The cells were sub-cultured every 2–3 days.

### Bone Marrow-Derived Macrophages (BMDMs)

Bone marrow was collected from femora of C57BL/6 mice. BMDMs were differentiated as reported previously (Kayagaki et al., 2013). In brief, bone marrow cells were differentiated in DMEM supplemented with 10% FBS and 20% (v/v) M-CSF-conditioned medium from L-929 fibroblasts for 6 days. BMDMs were then cultured in fresh DMEM complete medium overnight in 24-well plates at 1.5 × 10^5^ cells/well in 0.5 ml.

### Pyroptotic Assay

Cell death was measured by PI incorporation as described previously (Py et al., 2014). Cells were pre-treated with indicated concentration of piperine for 4 h in DMEM complete medium, and then primed with 500 ng/ml LPS for 4 h. Subsequently, the culture medium was replaced with Opti-MEM and indicated concentrations of ATP. Cell nuclei were revealed by Hoechst 33342 staining (5 μg/ml; staining for all cells) and PI (2 μg/ml; staining for pyroptotic cells) for 10 min. The cells were observed using a Zeiss Axio Observer D1 microscope equipped with a Zeiss LD Plan-Neofluar 20×/0.4 Korr M27 objective lens. Fluorescence images were captured with a Zeiss AxioCam MR R3 cooled CCD camera controlled with ZEN software (Carl Zeiss).

### Cytometric Bead Array

Soluble IL-1β was determined by cytometric bead array (CBA) using the Mouse IL-1β Flex Set (BD Biosciences, San Jose, CA, USA) according to the manufacturer’s instructions. Data were acquired using CELLQuest software on a flow cytometer (FACSCalibur; Becton Dickinson, Mountain View, CA, USA).

### Precipitation of Soluble Proteins in Supernatants

Proteins in culture supernatants (equal volume for each sample) were precipitated overnight with 7.2% trichloroacetic acid plus 0.15% sodium deoxycholate as previously described (Lin et al., 2016). The precipitates are lysed in equal volume of 1x sodium dodecyl sulfate-polyacrylamide gel electrophoresis (SDS-PAGE) loading buffer.

### Western Blot Analysis

Western blotting was performed as described previously (Lin et al., 2016) to detect proteins in the supernatants and cell lysates, respectively. In brief, total proteins were separated by SDS-PAGE and transferred onto a PVDF membrane (Amersham, RPN303F). After being blocked, the membranes were probed with indicated primary antibodies, followed by a HRP-conjugated goat anti-rabbit IgG or goat anti-mouse IgG. Bands were revealed by a BeyoECL Plus kit (Beyotime, P0018) and recorded on X-ray films (Kodak, 6535873). The densitometry of each band was quantified by FluorChem 8000 (Alpha Innotech; San Leandro, CA, USA).

### Bacterial Infection

C57BL/6 mice were acclimated for 1 week and intragastrically administered with piperine solution or vehicle (2% Tween-80 in PBS) once a day for 5 consecutive days. *E. coli* strain DH5α was grown in Luria Broth (LB) media and the bacterial cell density was determined using an ultraviolet-visible spectrophotometer (NanoDrop2000, Thermo Scientific). The colony-forming units (CFUs) corresponding to known cell densities were determined on LB agar plates. The mice were intraperitoneally (i.p.) injected with 2 × 10^9^ CFU/mouse of viable *E. coli* cells in 0.5 ml of PBS. After bacterial infection for indicated time lengths, the mice were sacrificed and peritoneal lavage fluids were collected with 1.5 ml PBS for IL-1β determination by CBA. The colon tissues were also collected for western blotting analysis. In a separate experiment, mouse sera were collected from retro-orbital venous blood and IL-1β in serum was assayed by CBA method.

### Statistical Analysis

All experiments were performed independently at least three times, with one representative experiment shown. Data are presented as mean ± standard deviation (SD). Statistical analysis was performed using Graphpad Prism 4.0 (GraphPad; San Diego, CA, USA). One-way analysis of variance (ANOVA) followed by Tukey *post hoc* test and unpaired Student’s *t*-test were used to analyze the statistical significance among multiple groups and between two groups, respectively. *P-*values < 0.05 were considered statistically significant.

## Results

### Piperine Inhibits ATP-Induced Pyroptosis in BMDMs

**FIGURE 1:**
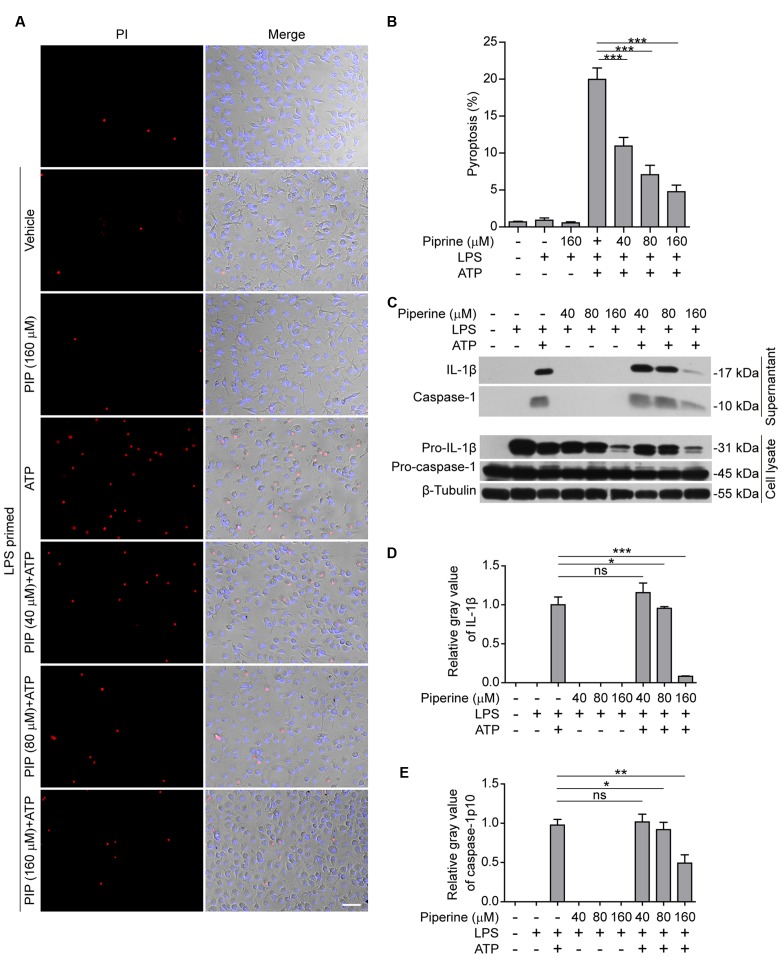
**Piperine inhibited ATP-induced pyroptosis in bone marrow-derived macrophages (BMDMs).** **(A)** Cells were pre-treated with graded concentrations of piperine for 4 h, followed by LPS (500 ng/ml) priming for 4 h (in the presence of piperine). Then the cells were stimulated with ATP (3 mM) for 30 min (in the absence of piperine and LPS). After these treatments, the cells were stained with 2 μg/ml propidium iodide (PI; red, staining dead cells) plus 5 μg/ml Hoechst 33342 (blue, staining all cells) for 10 min, and observed with fluorescent microscopy. One set of representative images of three independent experiments are shown. Bright-field images are also shown in merge. PIP, piperine. Scale bar, 50 μm. **(B)** PI-positive cells in five random fields (around 100 cells per field) were calculated and statistically analyzed. Data are shown as mean ± SD (*n* = 5). **(C)** Cells were treated as in **(A)**. The equal volumes of culture supernatants were collected and the proteins in the supernatants were precipitated overnight with 7.2% trichloroacetic acid plus 0.15% sodium deoxycholate. After centrifugation at 13,000 × *g* for 30 min, the pellets were lysed in equal volume of 1x SDS-PAGE loading buffer. Total proteins precipitated from equal volumes of supernatants were loaded for western blotting. The cells were lysed with 1× SDS-PAGE loading buffer. Equal amounts of proteins in the cell lysates were loaded for western blotting. β-Tubulin was recruited as a loading control in cell lysates. **(D,E)** Relative gray values of IL-1β **(D)** and caspase-1p10 **(E)** blots from respective supernatants were quantified. Data are shown as mean ± SD (*n* = 3). Statistical significance was analyzed by one-way ANOVA with Tukey *post hoc* test. ^∗^*P* < 0.05, ^∗∗^*P* < 0.01, ^∗∗∗^*P* < 0.001; ns, not significant.

Our previous work demonstrated that piperine administration significantly alleviated injury of internal organs and reduced mouse mortality in bacterial sepsis (Pan et al., 2015). As pyroptosis has been reported being causative of sepsis-induced organ damages, we explored whether piperine could suppress pyroptosis in LPS-primed macrophages upon ATP triggering. We firstly evaluated the effects of piperine on ATP-induced pyroptosis, which can be revealed by PI staining (Py et al., 2014). BMDMs were pretreated with piperine followed by LPS priming (in the presence of piperine) and were then triggered by ATP treatment (without LPS and piperine). The results showed that LPS priming did not induce pyroptosis in BMDMs, and piperine at a dose up to 160 μM was non-toxic to the cells during short periods of incubation (**Figures 1A,B**). Upon ATP triggering, however, the LPS-primed BMDMs rapidly underwent pyroptosis. Interestingly, piperine pretreatment significantly attenuated ATP-induced pyroptosis in a dose-dependent manner (**Figures 1A,B**).

It has been demonstrated that in LPS-primed macrophages ATP treatment activates NLRP3 inflammasome leading to pro-caspase-1 processing into active caspase-1p10; the latter in turn cleaves pro-IL-1β (31 kDa) to produce a 17-kDa IL-1β (mature form) while pyroptosis is induced and mature IL-1β is released from the pyroptotic cells rather than the living ones (Cullen et al., 2015). Therefore, the levels of mature IL-1β and active caspase-1p10 in cell culture supernatants are markers of inflammasome activation and pyroptosis. Consistent with these reports (Latz et al., 2013; Cullen et al., 2015), western blotting results showed that LPS priming greatly induced the expression of pro-IL-1β in the cells, whereas pro-caspase-1 was constitutively expressed in BMDM cells (**Figure 1C**). Without ATP treatment, both active caspase-1p10 and mature IL-1β were undetectable in the supernatants either treated with piperine or not; upon ATP triggering, they were significantly released into the supernatants, reflective of inflammasome activation and pyroptosis. Notably, piperine pretreatment dose-dependently suppressed the release of both active caspase-1p10 and mature IL-1β into the supernatants (**Figures 1C–E**). Together, these results indicated that piperine pretreatment could significantly attenuated ATP-induced pyroptosis, thus reducing IL-1β release, in LPS-primed BMDMs.

### Piperine Suppresses ATP-Induced Pyroptosis in J774A.1 Cells

**FIGURE 2:**
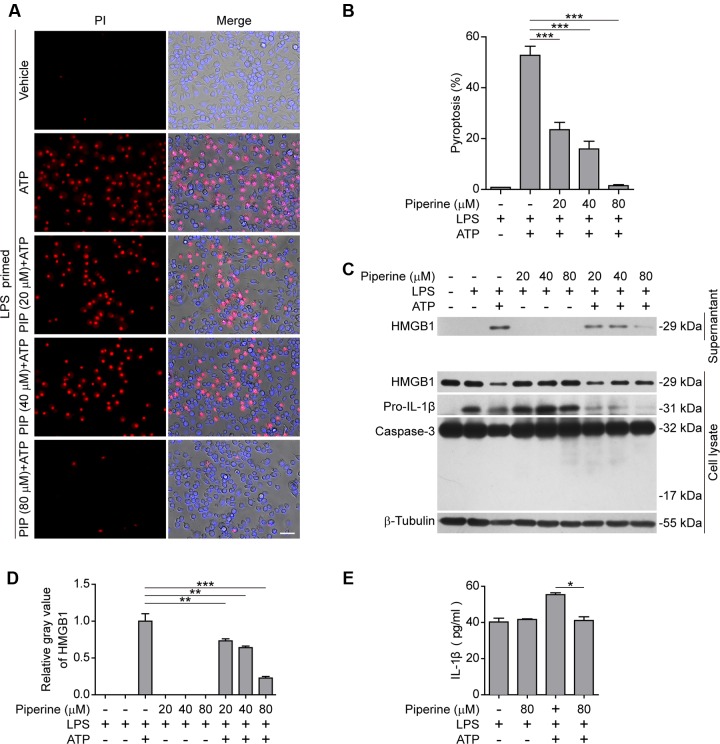
**Piperine suppressed ATP-induced pyroptosis in J774A.1 cells.** **(A)** Cells were pre-treated with graded doses of piperine for 4 h, followed by LPS (500 ng/ml) priming for 4 h (in the presence of piperine). Then the cells were stimulated with ATP (4 mM) for 1 h (in the absence of piperine and LPS). Pyroptotic cells were stained with 2 μg/ml propidium iodide (PI) plus 5 μg/ml Hoechst 33342 for 10 min, and observed by fluorescent microscopy. Bright-field images are also shown in merge. PIP, piperine. Scale bar, 50 μm. **(B)** PI-positive cells in 5 random fields (around 150 cells per field) were calculated for each group. Data are shown as mean ± SD (*n* = 5). **(C)** Cells were treated as in **(A)**. Proteins in cell lysates and culture supernatants were evaluated by western blotting. β-Tubulin was used as a loading control for cell lysates. **(D)** Relative gray values of HMGB1 blots from respective supernatants are shown as mean ± SD (*n* = 3). **(E)** IL-1β levels in the cell culture supernatants were evaluated by cytometric bead array (CBA) assay according to the manufacturer’s instructions. Data are shown as mean ± SD (*n* = 3). Statistical significance was analyzed by one-way ANOVA with Tukey *post hoc* test. *P < 0.05, **P < 0.01, ***P < 0.001.

Next, we examined the effects of piperine on ATP-induced pyroptosis in LPS-primed murine J774A.1 macrophage cell line. Piperine pretreatment dose-dependently suppressed ATP-induced pyroptosis as in BMDMs (**Figures 2A,B**). Consistently, ATP treatment induced the release of HMGB1, another danger signal molecule that is associated with inflammatory cell death (de Souza et al., 2012; Gdynia et al., 2016). Piperine pretreatment once again suppressed the effect of ATP on HMGB1 release (**Figures 2C,D**). As IL-1β release in the supernatants was hardly detectable by western blotting, we used a more sensitive CBA method (with a detection limit of ∼5 pg/ml) to measure soluble IL-1β levels. The result showed that piperine pretreatment significantly inhibited ATP-induced IL-1β release (**Figure 2E**). This is likely because piperine had directly suppressed the expression of pro-IL-1β in the cells treated with LPS plus ATP (**Figure 2C**, cell lysate). Apoptosis had not been involved in ATP-induced cell death, as caspase-3 cleavage (activation) was not observed in all groups (**Figure 2C**). Together, these results indicated that piperine treatment could suppress pyroptosis and reduced IL-1β and HMGB1 release in J774A.1 cells in response to ATP triggering.

Based on the above-mentioned results from both BMDMs and J774A.1 cells, piperine treatment could inhibit the pyroptosis in LPS-primed macrophages in response to ATP triggering.

### ATP-Induced AMPK Activation Is Suppressed by Piperine Treatment

**FIGURE 3:**
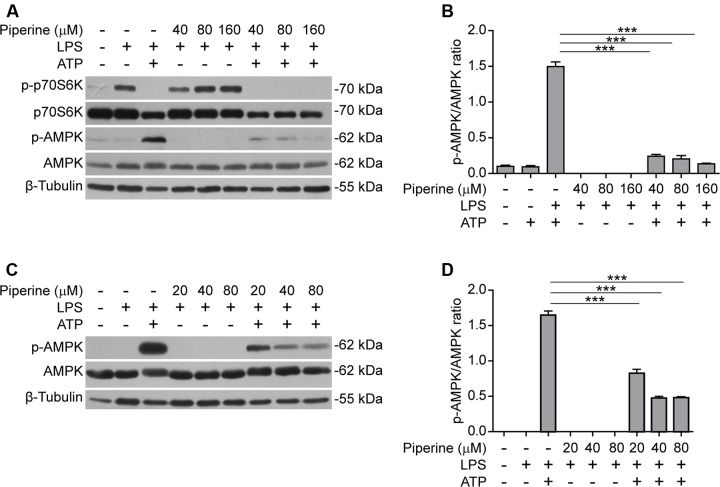
**Piperine attenuated ATP-induced AMPK activation in macrophages.** BMDMs **(A,B)** or J774A.1 cells **(C,D)** were pre-treated with graded concentrations of piperine for 4 h, followed by LPS (500 ng/ml) priming for 4 h before ATP (3 mM) stimulation for 30 min (BMDMs) or ATP (4 mM) stimulation for 1 h (J774A.1 cells), respectively, as mentioned in **Figure 1**. **(A,C)** The cells were lysed by 1x SDS-PAGE loading buffer and equal amounts of the total proteins in each sample were analyzed by western blotting with indicated antibodies. β-Tubulin was recruited as a loading control. **(B,D)** The densitometry ratios of p-AMPK relative to AMPK of BMDMs **(B)** or J774A.1 cells **(D)** were analyzed. Data are presented as mean ± SD (*n* = 3). Statistical significance was analyzed by one-way ANOVA with Tukey *post hoc* test. ***P < 0.001.

It has been shown that AMPK activity is suppressed by LPS, free fatty acid, and other inflammatory stimulators (Yang et al., 2010; Wen et al., 2011; Wang et al., 2016). However, AMPK signaling can be activated by bacterial infection (Bae H. B. et al., 2011) while the suppressed AMPK activity in LPS-primed macrophages can be dramatically activated (as reflected by its phosphorylation at Thr172) in response to ATP treatment (Bae H. B. et al., 2011; Moon et al., 2015). Consistent with these reports, we also observed that AMPK activity was suppressed by LPS but greatly increased in both LPS-primed BMDMs (**Figures 3A,B**) and J774A.1 cells (**Figures 3C,D**) upon ATP stimulation. Interestingly, ATP-induced AMPK activation could be markedly suppressed by piperine pretreatment (**Figures 3A–D**). Consistent with our previous study (Pan et al., 2015), piperine could increase the phosphorylation of p70S6K, indicative of enhanced mTORC1 activity, in macrophages (**Figure 1A**). In line with the activation of AMPK upon ATP treatment, the mTORC1 activity was sharply suppressed by ATP. These results in conjunction with previous studies suggested that AMPK signaling may regulate cell survival and death under inflammatory stresses. Our results also suggested that piperine might inhibit ATP-induced pyroptosis by suppressing AMPK activation.

### Piperine Attenuates Pyroptosis by Suppressing AMPK Signaling

**FIGURE 4:**
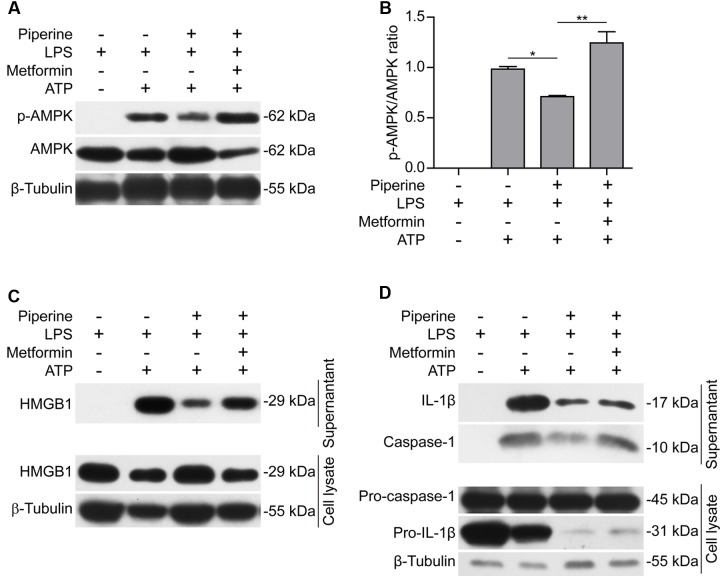
**Metformin, an AMPK agonist, counteracted the effect of piperine on suppressing ATP-induced AMPK activation and inflammatory mediator release.** J774A.1 cells **(A–C)** were pre-treated with 80 μM piperine and BMDMs **(D)** were pre-treated with 160 μM piperine for 4 h. Without being washed out of piperine, these cells were primed with LPS (500 ng/ml) for 4 h. Next, the cells were treated with metformin (1 mM) for 1 h (in the absence of piperine and LPS). Finally, in the presence of metformin, the BMDMs were stimulated with 3 mM ATP (final concentration) for 30 min while the J774A.1 cells were treated with 4 mM ATP (final concentration) for 1 h. After the cells were lysed with 1x SDS-PAGE loading buffer, protein levels were evaluated by western blotting **(A,C,D)**. β-Tubulin was used as a loading control for cell lysates. **(B)** The densitometry ratios of p-AMPK relative to AMPK in the blots of **(A)** were analyzed by FluorChem 8000 (Alpha Innotech). Data are presented as mean ± SD (*n* = 3). Statistical significance was analyzed by one-way ANOVA with Tukey *post hoc* test. *^∗^P* < 0.05, ^∗∗^*P* < 0.01.

**FIGURE 5:**
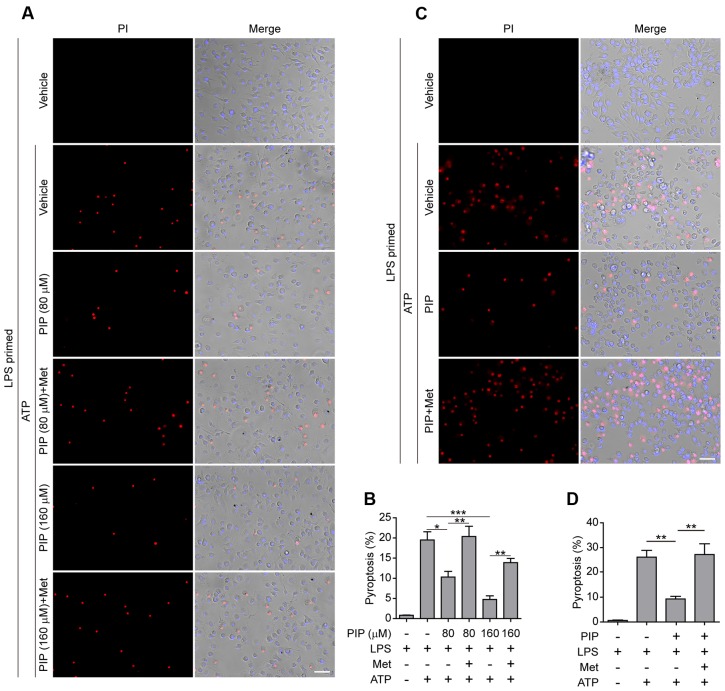
**Metformin reversed piperine’s effect on suppression of pyroptosis in macrophages treated with ATP.** BMDMs **(A,B)** or J774A.1 cells **(C,D)** were pre-treated with indicated concentrations of piperine (BMDMs) or 80 μM piperine (J774A.1) for 4 h, and then primed with LPS (500 ng/ml) for 4 h in the presence of piperine. After washing out of piperine and LPS, the cells were then treated with metformin (1 mM) for 1 h followed by 3 mM ATP for 30 min (BMDMs) or 4 mM ATP for 1 h (J774A.1 cells) in the presence of metformin. The pyroptotic cell ratios in BMDMs **(B)** or J774A.1 cells **(D)** were evaluated as described in **Figure 1**. Data are presented as mean ± SD (*n* = 5). Statistical significance was analyzed by one-way ANOVA with Tukey *post hoc* test. ^∗^*P* < 0.05, ^∗∗^*P* < 0.01, ^∗∗∗^*P* < 0.001. PIP, piperine; Met, metformin.

Having found that AMPK activity was associated with piperine-mediated suppression of pyroptosis in macrophages in response to ATP stimulation, we next investigated whether such suppressive effects of piperine could be counteracted by boosting AMPK signaling with metformin, a well-known AMPK agonist (Su and Dai, 2016). As expected, metformin could reverse piperine-mediated suppression of AMPK activity in macrophages in response to LPS and ATP stimulation (**Figures 4A,B**). Notably, metformin treatment counteracted piperine-mediated suppression of HMGB1 release from J774A.1 cells (**Figure 4C**, supernatant). Metformin treatment also counteracted the inhibitory effect of piperine on ATP-induced active caspase-1p10 release from BMDMs, but the reduced IL-1β release induced by ATP in the presence of piperine was not completely restored by metformin. This was likely due to that the expression of pro-IL-1β was greatly suppressed by piperine of 160 μM in LPS+ATP-treated BMDMs (**Figure 4D**, cell lysate). Consistent with these results, metformin fully reversed the effect of 80 μM piperine on suppressing ATP-induced pyroptosis in BMDMs (**Figures 5A,B**) and J774A.1 cells (**Figures 5C,D**), while partially reversing the inhibitory effect of 160 μM piperine on ATP-induced pyroptosis in BMDMs (**Figure 5B**) probably due to the robust inhibitory effect of this high-dose piperine. These results demonstrated that piperine pretreatment protected LPS-primed macrophages from ATP-induced pyroptosis by at least partly suppressing AMPK activation.

### Piperine Administration Reduces Systemic IL-1β Release in Mouse Bacterial Sepsis

**FIGURE 6:**
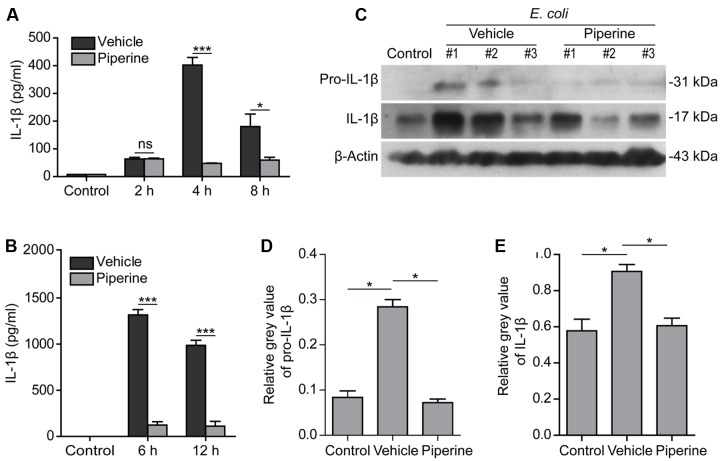
**Piperine administration reduced systemic IL-1β release in mouse bacterial sepsis.** Piperine was suspended in 2% Tween-80 in PBS at a concentration of 1 mg/ml. C57BL/6 mice (5 per group for each time point) were administered with piperine (20 mg/kg) or vehicle via gavage once a day for five consecutive days and injected intraperitoneally (i.p.) with viable *E. coli* (2 × 10^9^ CFU/mouse, in 0.5 ml PBS) 1 h after the last gavage. Peritoneal lavage fluids were collected with 1 ml of PBS. Serum was isolated from retro-orbital blood sample. Soluble IL-1β was evaluated by CBA (cytometric bead array) according the manufacturer’s instruction. **(A)** The levels of soluble IL-1β in peritoneal lavage fluids at 2, 4, and 8 h post bacterial infection. Data are presented as mean ± SD (*n* = 5). **(B)** The serum levels of IL-1β at 6 and 12 h post bacterial infection. Data are presented as mean ± SD (*n* = 5). The significance was analyzed with unpaired Student’s *t*-test. **(C)** A middle colon section with a length of 1 centimeter was cut from each mouse and immediately lysed by grinding with 500 μl 2x SDS-PAGE loading buffer. Western blotting was used to detect the expression of pro-IL-1β and IL-1β in the colonic cell lysates. β-Actin was used as a loading control. Blots of three mice from each group are shown. **(D,E)** Relative gray values of pro-IL-1β **(D)** and IL-1β in **(E)** were analyzed by one-way ANOVA with Tukey *post hoc* test. Data are shown as mean ± SD (*n* = 3). ^∗^*P* < 0.05, ^∗∗∗^*P* < 0.001.

Previously, we have demonstrated that piperine administration *in vivo* can protect mice from bacterial infection and that the mice having received piperine administration showed less mortality and histological injuries in their liver and colon as compared to the vehicle group (Pan et al., 2015). In this study, we further investigated whether piperine administration reduced IL-1β release in C57BL/6 mice upon bacterial infection. The results showed that bacterial infection induced IL-1β release into the peritoneal lavage fluids and blood in a time-dependent manner (**Figures 6A,B**). Piperine administration significantly reduced IL-1β levels both in the peritoneal lavage fluids and serum (**Figures 6A,B**), suggesting that bacterial-induced systemic inflammation and pyroptosis was suppressed by piperine. Western blotting also demonstrated that bacterial infection increased both pro-IL-1β and mature IL-1β (17 kDa) expression in the colonic tissues, suggesting inflammasome activation in the colon, which might be infected by bacteria injection in the peritoneal cavity. Piperine administration greatly suppressed the expression of pro-IL-1β and IL-1β in the colonic tissues (**Figures 6C–E**), which was likely to reduce the secretion of IL-1β from the colon. Altogether, these results suggested that piperine administration could inhibit systemic inflammatory responses during bacterial sepsis, probably through suppressing pyroptosis of activated immune cells including macrophages.

## Discussion

It has been shown that piperine exhibits anti-septic effects in a mouse model of bacterial sepsis probably by boosting the functions of peritoneal resident macrophages through the upregulation of mTORC1 signaling (Pan et al., 2015). However, this may not thoroughly explain why piperine has alleviated the damages to internal organs upon bacterial infection. In this study, we further demonstrated that piperine treatment decreased ATP-induced pyroptosis and IL-1β release in LPS-primed macrophages *in vitro*, and suppressed IL-1β expression and release *in vivo* in the same murine septic model. These data strongly suggest that piperine may have suppressed pyroptosis *in vivo* in the circumstance of bacterial infection, thereby protecting mice from septic death by reducing systemic inflammatory responses as well as organ damages.

Sepsis is a worldwide medical problem, since the pathological progression from systemic infection to septic shock or death can be very fast and there are at present no therapeutic drugs to prevent it (Dellinger et al., 2013). One consequence of sepsis is multiple organ failure in the patients. Several cell death types, including apoptosis, necrosis and pyroptosis, have been implicated in this process (Aziz et al., 2014). However, recent studies tend to agree that pyroptosis is the major cause of organ injury from sepsis (Wree et al., 2014; Kayagaki et al., 2015). Indeed, hepatic pyroptosis due to hyperactive NLRP3 inflammasome has been found to contribute to liver damage (Wree et al., 2014). Consistent with this concept, mice with *caspase-1/-11* or *gasdermin D* gene deletion, thus lacking inflammasome activation and pyroptosis, are resistant to endotoxin-induced sepsis (Kayagaki et al., 2011, 2015). In further support of this, inhibition pyroptosis by neutralization of LPS with antimicrobial peptide LL-37 significantly protects mice from cecal ligation and puncture (CLP)-induced death (another commonly used septic mouse model) (Hu et al., 2016). Therefore, preventing pyroptosis during bacterial infection should alleviate sepsis-associated multiple organ failure and pathological process.

Pyroptosis can be induced by ATP treatment in LPS-primed macrophages *in vitro*. Such a cellular pyroptotic model may represent a severe circumstance of *in vivo* infection, since ATP can be released by both bacteria and host cells during bacterial infection (Mempin et al., 2013; Ren et al., 2014). In this study, we demonstrated that piperine pretreatment suppressed ATP-induced pyroptosis in LPS-primed macrophages *in vitro*, which was evidenced by the reduction of soluble mature IL-1β, active caspase-1p10 and HMGB1 in the supernatants of piperine-pretreated macrophages. One reason for the reduced soluble mature IL-1β levels might be due to piperine-mediated suppression of pyroptosis. A second reason was likely due to that high doses of piperine significantly reduced the pro-IL-1β level in LPS-primed BMDMs irrespective of ATP treatment (**Figure 1C**) as well as in LPS-activated J774A.1 cells in the presence of ATP (**Figure 2C**). The precise mechanism underlying reduced pro-IL-1β levels is unknown but it is probably that a high dose of piperine may influence the expression of pro-IL-1β at transcriptional and translational levels or at post-translational levels. Importantly, piperine may also suppressed *in vivo* pyroptosis in the murine bacterial septic model, as reflected by reduced mature IL-1β levels in serum and infected sites. Consistent with the *in vitro* observation, piperine administration also down-regulated the pro-IL-1β levels in the colon, which might also contribute to reduced mature IL-1β levels in this tissue (**Figures 6C,D**). As pyroptosis has a critical role in septic shock (Wree et al., 2014; Kayagaki et al., 2015), reduced pyroptosis by piperine may diminish the injury of organs. Indeed, piperine administration *in vivo* alleviated injuries of internal organs and reduced mouse mortality upon bacterial infection (Pan et al., 2015). This is consistent with a previous study showing that piperine can inhibit LPS-induced endotoxin shock, although there was no reduction in IL-1β levels in serum (detected at 3 h post LPS injection) of mice treated with piperine intraperitoneally (Bae et al., 2010). The discrepancy between their results and ours may be due to the differences of animal models and drug administration routes. In our study, the mice were directly infected with viable bacteria and piperine was given intragastrically. Moreover, the release of IL-1β in our experimental model was time-dependent and we observed varied serum IL-1β levels in the vehicle and piperine groups at 6 and 12 h. It is unclear whether piperine also suppressed serum IL-1β levels in LPS-inoculated mice in a time-dependent manner.

Chronic inflammations including autoimmune diseases are associated with inflammasome activation (Yang and Chiang, 2015; Zhang et al., 2016). Negative regulation of the inflammasome activation is believed to ameliorate these diseases (Vande Walle et al., 2014), whereas release of IL-1β, IL-18, HMGB1 or uric acid may induce or aggravate disease symptoms (Lamkanfi et al., 2011; Yang and Chiang, 2015). Piperine has been reported to prevent chronic inflammatory diseases, including epilepsy (Pal et al., 2011) and arthritis (Umar et al., 2013). Some studies have indicated or implied that piperine alleviates chronic inflammatory diseases (such as diabetic nephropathy (Samra et al., 2016) and arthritis (Ying et al., 2013)) by suppressing inflammasome activation and thus inhibiting IL-1β release. Consistent with our study, one recent report indicated that piperine inhibits periodontitis in a rat model at a dose of 100 mg/kg (Dong et al., 2015). This is likely due to its inhibitory effect on the expression of IL-1β (or pro-IL-1β), matrix metallopeptidase (MMP)-8 and MMP-13 in the local gingiva tissues. However, it is unclear whether piperine suppresses pyroptosis and IL-1β release in the gingiva tissues of the periodontitis model.

It is of importance to uncover the underlying mechanism by which piperine suppressed pyroptosis and IL-1β release. Our previous study has demonstrated that piperine treatment promotes amino acid metabolism to enhance mTORC1 signaling (Pan et al., 2015). Therefore, we investigated whether piperine influenced the mTORC1 signaling in LPS-primed macrophages upon ATP stimulation. Our results demonstrated that the mTORC1-p70S6K pathway was greatly activated in macrophages by LPS priming, but was completely suppressed by ATP treatment (**Figure 3A**). Consistent with a previous observation (Moon et al., 2015), our study also demonstrated that ATP treatment greatly induced the activation of AMPK. Both mTORC1 and AMPK are key regulators of energy metabolism. Under nutrition or other stresses, AMPK is activated (Hardie, 2011b). Multiple cellular activities can be regulated by AMPK activation (Hardie, 2011a). For example, AMPK phosphorylates and inhibits the activity of acetyl-CoA carboxylase (ACC), an enzyme responsible for the catalysis of acetyl-CoA into malonyl-CoA during the β-oxidation of fatty acids (Ha et al., 1994; Hardie and Pan, 2002). It also inhibits the activity of mTORC1 by phosphorylation of TSC2 (a suppressor of mTORC1) and Raptor [one member of mTORC1 (Inoki et al., 2003; Gwinn et al., 2008)]. This leads to a general inhibition of protein translation (Hardie, 2011a; Thoreen et al., 2012). In the innate immunity, AMPK activation in macrophages and neutrophils enhances their phagocytosis ability against pathogens (Bae H. B. et al., 2011). Interestingly, AMPK signaling can be suppressed by LPS, fatty acid and other inflammatory stimulators (Yang et al., 2010). However, it can be dramatically re-activated when a second DAMP signal (such as ATP) is added, accompanied by inflammasome assembly (Moon et al., 2015). Under this circumstance, the cells resort to HK1-dependent glycolysis upon inflammasome activation (Moon et al., 2015). This means that there is a sharp switch of energy metabolism from oxidative phosphorylation to glycolysis during the process of inflammasome activation (including the progression phases from LPS priming to ATP stimulation). As mTORC1 can stabilize the hypoxia-induced factor-1α (HIF-1α), it favors cell survival in LPS-primed macrophages (Gilroy and Yona, 2015). However, AMPK activation, through suppressing mTORC1 activity, decreases the level of HIF-1α and makes the cells undergo death processes (Jiang et al., 2001; McGettrick and O’Neill, 2013). Although piperine treatment seemed unable to reverse the suppression of mTORC1 activity (as reflected by p70S6K phosphorylation) by ATP, it significantly suppressed ATP-induced AMPK activation (**Figure 3A**). This implies that piperine attenuated the inhibitory activity of AMPK on mTORC1 signaling upon ATP treatment. In addition, metformin (acting as an AMPK agonist) could counteract the effect of piperine on reducing ATP-induced pyroptosis. These results suggested that suppressing AMPK activation by piperine (or by other AMPK inhibitors) during systemic bacterial infection may be helpful for preventing the pathological development of sepsis. However, more research is warranted to reveal whether piperine ameliorates other inflammatory diseases, including epilepsy, obesity, arthritis and ulcer by the common mechanism of inflammasome suppression as in bacterial sepsis.

Piperine is the major plant alkaloid in pepper, a daily used food seasoning. Therefore, it is believed to be low toxic to mammalian cells and human body. Indeed, our preliminary experiments showed that piperine had no overt toxicity to mouse BMDMs at a dose up to 160 μM *ex vivo* and to mice at a dose up to 40 mg/kg/day *in vivo*. Supporting this, a previous study showed that piperine has no oral acute toxicity on mice at a dose up to 5000 mg/kg (Gupta et al., 2015). This means that piperine may be safe for humans at doses above tens of milligrams based on translating dosages from animal models to human clinical trials (Blanchard and Smoliga, 2015). Although there are currently no clinical trials testing piperine for bacterial sepsis, other clinical trials have recruited piperine at doses of 20–25 mg each time or per day^1^. Another clinical trial had used piperine locally at 1 and 0.15 mM to evaluate the effect of piperine in patients with oropharyngeal dysphagia^2^. Thus, the *in vitro* and *in vivo* doses used in this study have relevance for translating into human study.

In summary, we revealed that piperine treatment could significantly reduce ATP-induced pyroptosis in macrophages probably through the suppression of AMPK signaling. In a murine bacterial sepsis model, piperine administration sharply decreased systemic IL-1β levels, suggestive of suppression of systemic inflammation and pyroptosis. Our data highlight that piperine may act as a suppressant of pyroptosis to exhibit its therapeutic effects on bacterial sepsis and other inflammatory disorders, which deserves further clinical investigation.

## Author Contributions

Y-DL, W-JB, C-GL, L-HX, and H-XW performed the experiments. D-YO and X-HH designed the research. Y-DL, C-GL, and HP analyzed the data. D-YO, X-HH, and Y-DL wrote the paper. All authors approved the final version of the manuscript.

## Conflict of Interest Statement

The authors declare that the research was conducted in the absence of any commercial or financial relationships that could be construed as a potential conflict of interest.

## Footnotes

1 http://clinicaltrialsfeeds.org/clinical-trials/results/term=Piperine

2 https://clinicaltrials.gov/show/NCT01383694
